# Comparing family-based rare variant association tests for dichotomous phenotypes

**DOI:** 10.1186/s12919-016-0027-8

**Published:** 2016-10-18

**Authors:** Longfei Wang, Sungkyoung Choi, Sungyoung Lee, Taesung Park, Sungho Won

**Affiliations:** 1 Interdisciplinary Program in bioinformatics, Seoul National University, Seoul, 151-742 Korea; 2 Department of Statistics, Seoul National University, Seoul, 151-742 Korea; 3 Department of Public Health Science, Seoul National University, Seoul, 151-742 Korea; 4 Institute of Health Environment, Seoul National University, Seoul, 151-742 Korea

## Abstract

**Background:**

It has been repeatedly stressed that family-based samples suffer less from genetic heterogeneity and that association analyses with family-based samples are expected to be powerful for detecting susceptibility loci for rare disease. Various approaches for rare-variant analysis with family-based samples have been proposed.

**Methods:**

In this report, performances of the existing methods were compared with the simulated data set provided as part of Genetic Analysis Workshop 19 (GAW19). We considered the rare variant transmission disequilibrium test (RV-TDT), generalized estimating equations-based kernel association (GEE-KM) test, an extended combined multivariate and collapsing test for pedigree data (known as Pedigree Combined Multivariate and Collapsing [PedCMC]), gene-level kernel and burden association tests with disease status for pedigree data (PedGene), and the family-based rare variant association test (FARVAT).

**Results:**

The results show that PedGene and FARVAT are usually the most efficient, and the optimal test statistic provided by FARVAT is robust under different disease models. Furthermore, FARVAT was implemented with C++, which is more computationally faster than other methods.

**Conclusions:**

Considering both statistical and computational efficiency, we conclude that FARVAT is a good choice for rare-variant analysis with extended families.

## Background

It has been reported that rare variants may be functionally more related to diseases than common variants [1–3]. However, in spite of their importance, individual tests of rare variants lead to large false-negative findings as the marginal effect of a rare variant cannot be detected unless very large samples are available. Alternatively, the collapsed rare allele counts or variance inflations for multiple rare variants in a gene can be compared between affected and unaffected individuals, and several burden and variance component methods have successfully identified the genetic association of rare variants [3, 4].

In spite of these successful findings, the analysis with population-based samples suffers from genetic heterogeneity. The common-disease rare-variant hypothesis assumes that there are multiple rare causal variants, and it has often been expected that rare causal variants may not be shared between affected individuals. Consequently, rare variant association analysis with population-based samples might suffer from the genetic heterogeneity between affected individuals, and various analysis strategies, such as selecting individuals with extreme phenotypes, have been proposed to minimize genetic heterogeneity [5, 6]. In particular, individuals in a family are genetically more homogeneous, and affected family members have an increased chance to share the same causal variants. In this context, the importance of family-based samples has been repeatedly stressed, and several family-based approaches have been proposed [7–10].

In this report, we compare the performance of existing rare variant association methods for family-based samples using Genetic Analysis Workshop 19 (GAW19) simulated data. We considered 5 different methods for dichotomous phenotypes: the rare variant transmission disequilibrium test (RV-TDT) [11], generalized estimating equations based kernel association (GEE-KM) test [9], an extended combined multivariate and collapsing test for pedigree data (Pedigree Combined Multivariate and Collapsing [PedCMC]) [10], gene-level kernel and burden association tests with disease status for pedigree data (PedGene) [8], and the family-based rare variant association test (FARVAT) [12]. The family-based association test (FBAT) [13] was not included in our power comparison, but its power is expected to be similar to that of the RV-TDT because both are based on transmission disequilibrium tests. These methods were utilized to identify causal genes for hypertension, and results were compared with regard to their statistical and computational efficiency. Our results showed that PedGene and FARVAT are usually the most statistically efficient, and with regards to the computational efficiency, FARVAT is the most efficient.

## Methods

### Rare variant transmission disequilibrium test

RV-TDT [11] is an extension of the transmission disequilibrium test (TDT) to analyze parent–child trio data for rare-variant associations, which can adequately control for population admixture. RV-TDT is implemented with C and can calculate five commonly used methods: 1) TDT-CMC: extension of Combined Multivariate and Collapsing (CMC) [14]; 2) TDT-BRV: extension of Burden of Rare Variants (BRV) [15]; 3) TDT-VT-BRV: extension of Variable Threshold (VT) [3] with BRV coding; 4) TDT-VT-CMC: extension of VT with CMC coding; 5) TDT-WSS: extension of Weighted Sum Statistic (WSS) [16].

### Generalized estimating equations-based kernel association

Wang et al. [9] extended the optimal sequence kernel association test (SKAT-O) method [17] to family-based samples with generalized estimating equations (GEEs). GEE-KM can handle both continuous and discrete phenotypes, and the phenotypic correlation among family members is taken into account with an empirical correlation matrix. GEE-KM can adjust for the effect of covariates, and was implemented in the gskat R package.

### Pedigree combined multivariate and collapsing

PedCMC [10] was proposed as an extension of the combined multivariate and collapsing test [14] for population-based samples to family-based samples. The genotypes for rare variants in each gene are coded as either 0 or 1, according to the presence of rare alleles, and sums of coded genotypes are compared between affected and unaffected individuals.

### PedGene

Schaid et al. [8] proposed burden and kernel statistics for extended families, and it was implemented in the PedGene R package. The kernel statistic is a variance component test and is more efficient than a burden test if there are both protective and deleterious variants in a gene.

### Family-based rare variant association test

Choi et al. [12] proposed FARVAT based on the quasi-likelihood. FARVAT provides burden-type, C-alpha-type and optimal sequence kernel association test (SKAT-O)–type statistics. SKAT-O–type statistics are derived by weighting burden-type and C-alpha-type statistics with an optimal weight [3]. FARVAT was implemented with C++. The C-alpha statistic corresponds to the kernel test in PedGene.

### Data

We focused on the sequencing data in a pedigree-based sample from GAW19. Individuals with systolic blood pressure (SBP) less than 140 or diastolic blood pressure (DBP) greater than 90 were assigned to be affected by hypertension. Genotypes for 959 individuals imputed from 464 sequenced subjects were used in our analysis, and we considered rare variants whose minor allele frequencies were less than 0.05. Rare variants were annotated with high-, moderate-, and low-risk effect by using SnpEff software [18], and those variants were used for gene-set analysis. The set file included 58,969 single-nucleotide polymorphisms (SNPs) for 7210 genes, which was used to evaluate the statistical validity for all methods. We focused on the hypertension status, and the analysis results from 200 simulated data were compared.

## Results and discussion

### Empirical sizes

**Fig. 1:**
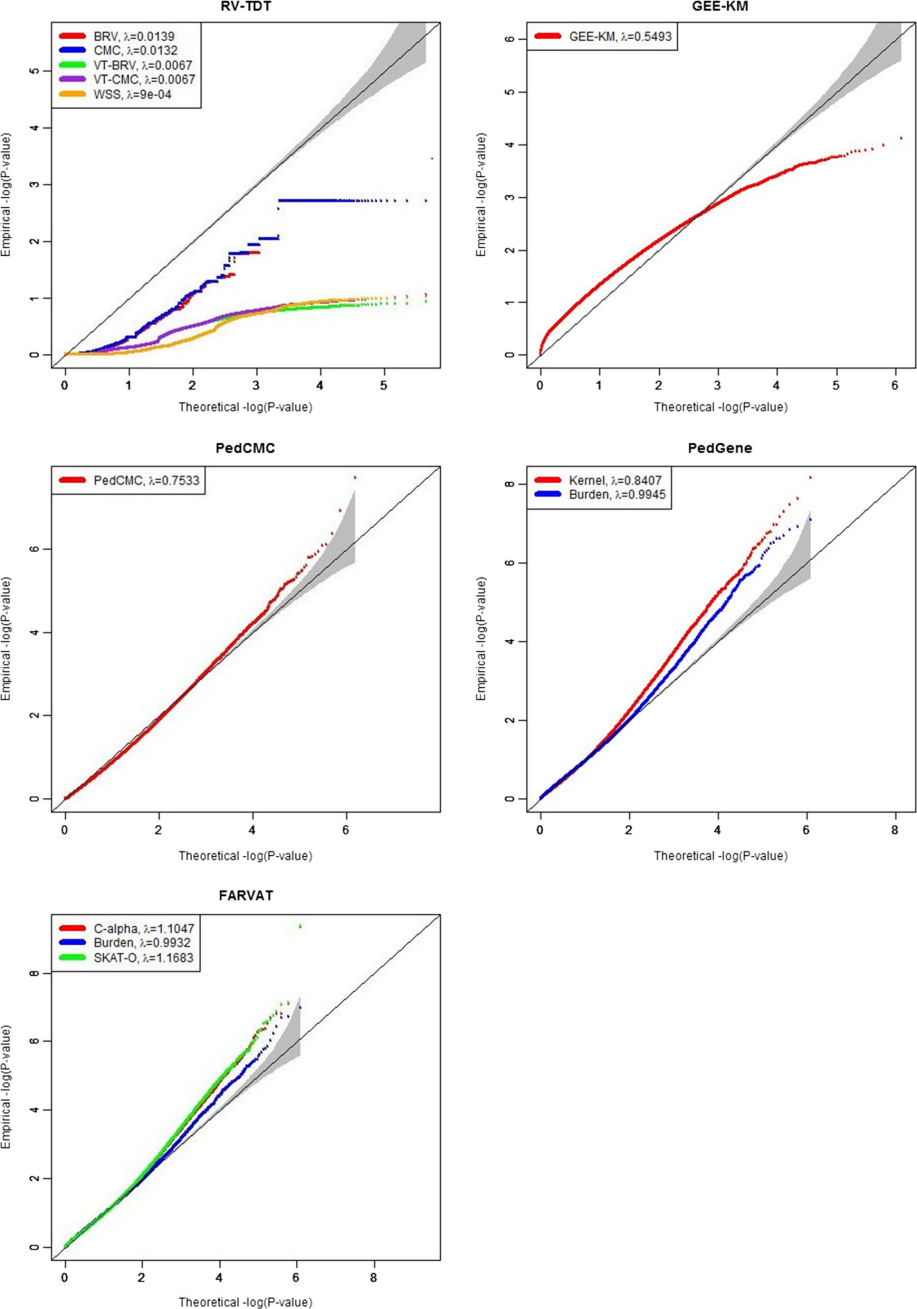
Quantile–quantile (Q-Q) plots for all methods

For the evaluation of statistical validity, the empirical type I error estimates for all methods were calculated at various significance levels with 200 replicates. We used Q1 as the phenotype and converted it to binary phenotype with prevalence 22.6 %. There were 7210 genes in each replicate, and thus 71,442,000 *p* values were utilized to calculate the empirical sizes. Table 1 shows the empirical type 1 error estimates for all methods at various significance levels. Results showed that RV-TDT methods have obvious deflated type 1 error rates, and GEE-KM test has an inflated type 1 error rate. The other methods seem to preserve the nominal significance levels. Figure 1 shows quantile-quantile (Q-Q) plots, and the estimated genomic inflation factor, λ, for all methods. All results from 200 replicates were combined and were utilized to build Q-Q plots. Figure 1 shows that PedCMC, PedGene, and FARVAT seem to control the type 1 error rates well, but the estimated inflation factors of C-alpha and SKAT-O tests from FARVAT show some inflation. Q-Q plots of results from RV-TDT show obvious deflation, and the extent of deflation is substantial for VT-BRV, VT-CMC, and WSS. Statistics in RV-TDT handle only trio data, and it may be the main reason of the deflation. The results for GEE-KM appear to be invalid. GEE-KM used the sandwich estimators for the correlation matrix between family members, and its results can be biased if the number of repeated measurement is not sufficient [19]. In our case, family sizes are different, and thus the sandwich estimator was estimated with a single observation, which may be the main reason of the invalid results from GEE-KM.

**Table 1:** Empirical sizes calculated with 7210 genes from 200 replicates

α	RV-TDT	GEE-KM	PedCMC	PedGene	FARVAT
	CMC/BRVVT-BRV/VT-CMC/WSS			Kernel/burden	C-alpha/burden/SKAT-O
0.1	0.0108/0.0130/0/0/0	0.2137	0.0714	0.0895/0.0879	0.0865/0.0888/0.0864
0.05	0.0040/0.0040/0/0/0	0.1050	0.0357	0.0490/0.0433	0.0445/0.0434/0.0450
0.01	0.0009/0.0009/0/0/0	0.0163	0.0079	0.0141/0.0098	0.0112/0.0092/0.0115
0.005	0.0004/0.0004/0/0/0	0.0066	0.0043	0.0086/0.0056	0.0065/0.0050/0.0068
0.001	0/0/0/0/0	0.0006	0.0011	0.0029/0.0017	0.0020/0.0013/0.0021

### Empirical power

Genes with the top 6 largest effects on both simulated SBP and DBP were selected to evaluate the empirical powers for all methods. Rare variants in the selected genes with causal effects on SBP and DBP are all included for each gene-set file, and a certain number of rare variants with no effect in each gene were randomly selected to make the proportion of causal variants 10, 25, and 50 %. We found that the results for 25 % are similar to those for 10 and 50 %, and we only presented results for 10 and 50 % in Table 2. In addition, the results for RV-TDT are all zero, and thus are not presented in Table 2. Table 2 shows that the FARVAT method seems to be the most efficient and it is followed by PedGene, though the differences are small. In particular, the statistical efficiency of burden and C-alpha/kernel statistics depends on the unknown disease model, and the empirical power estimates of the SKAT-O–type FARVAT are usually close to the most efficient approaches. Therefore, the robust statistic against unknown genetic distributions of causal variants is uniquely provided by FARVAT. Power when 50 % of rare variants are causal is less than the power when 10 % are causal, which might be attributed to insufficient number of replicates. Overall, we can conclude that FARVAT and PedGene are usually the most efficient methods for the rare-variant analysis with extended families, and the SKAT-O test provided by FARVAT is a robust method under different disease models.

**Table 2:** Empirical power for the top 6 causal genes affecting both simulated SBP and DBP at the 0.05 significance level

GENE	Proportion of causal variants	GEE-KM	PedCMC	PedGene		FARVAT		
				Kernel	Burden	C-alpha	Burden	SKAT-O
*MAP4*	10 %	0.005	0.110	0.065	0.015	0.160	0.055	0.105
	50 %	0.075	0.165	0.190	0.485	0.270	0.545	0.435
*NRF1*	10 %	0.010	0.000	0.005	0.010	0.015	0.020	0.020
	50 %	0.005	0.020	0.115	0.065	0.070	0.015	0.055
*TNN*	10 %	0.045	0.005	0.005	0.005	0.005	0.010	0.005
	50 %	0.085	0.020	0.025	0.020	0.025	0.025	0.025
*LEPR*	10 %	0.010	0.075	0.005	0.045	0.010	0.055	0.030
	50 %	0.000	0.010	0.020	0.010	0.020	0.020	0.010
*FLT3*	10 %	0.000	0.245	0.440	0.160	0.505	0.255	0.450
	50 %	0.035	0.040	0.525	0.410	0.450	0.395	0.425
*ZNF443*	10 %	0.215	0.005	0.090	0.090	0.060	0.065	0.050
	50 %	0.185	0	0.190	0.045	0.125	0.010	0.075
Mean	10 %	0.048	0.073	0.102	0.054	0.126	0.077	0.110
	50 %	0.064	0.043	0.178	0.173	0.160	0.168	0.171
Median	10 %	0.010	0.040	0.035	0.030	0.038	0.055	0.040
	50 %	0.055	0.020	0.153	0.055	0.098	0.023	0.065

Furthermore, we compared other features of each method, such as computational time, and the summary is provided in Table 3. According to Table 3, GEE-KM is a unique statistic for prospective design, and it compares the phenotypic distributions for each coded genotype whereas the other methods compare genetic distributions between affected and unaffected individuals. GEE-KM is also a unique approach that can adjust effect of covariates with a logistic link function. PedGene and FARVAT use the linear mixed model to adjust the effect of covariates. Work by Crowder [20, 21] suggests that the choice of a linear mixed model often work reasonably well for dichotomous phenotypes. The SKAT-O–type statistic, which is robust against the distribution of genetic effects, is uniquely provided by FARVAT. Last, in our analyses, we used Intel Xeon CPU E5-2620 0 @ 2.00GHz with 10 node and 80 gigabyte memory, and computational times to complete all analyses is shown. The computational time difference is related with the programming language, and software implemented with C/C++ is usually fast [17]. Table 3 shows that FARVAT is the most computationally efficient.

**Table 3:** Summary for all methods

Method	Design	Phenotype	Burden	C-alpha	SKAT-O	Covariate	Language	Computing time (hour)
RV-TDT	Retrospective	Binary					C	20
GEE-KM	Prospective	Binary/Continuous	√	√		√	R	40
PedCMC	Retrospective	Binary	√				C	1.7
PedGene	Retrospective	Binary	√	√			R	40
FARVAT	Retrospective	Binary	√	√	√		C	1.7

## Conclusions

In this report, we evaluated several FBATs for detecting rare variants using GAW19 data. We found that FARVAT and PedGene usually provide similar statistical efficiency, and recommend the SKAT-O–type statistic provided by FARVAT because its power has been robust under various disease models. In addition, FARVAT was implemented with C++, and we found that it was computationally fast. Furthermore, it can load various input file formats, and provides additional information about minor allele counts. Therefore, we can conclude that FARVAT is a good strategy for rare-variant analysis with extended families in terms of both computational and statistical efficiency.
